# Tricuspid transcatheter edge-to-edge repair in local anaesthesia guided by three-dimensional intracardiac echocardiography: a case report

**DOI:** 10.1093/ehjcr/ytag466

**Published:** 2026-06-22

**Authors:** Lukas Ley, Anna Sannino, Mario Kasner, Vasileios Exarchos, Ulf Landmesser, Markus Reinthaler, Fabian Barbieri

**Affiliations:** Deutsches Herzzentrum der Charité, Department of Cardiology, Angiology and Intensive Care Medicine, Campus Benjamin Franklin, Hindenburgdamm 30, Berlin 12203, Germany; Charité—Universitätsmedizin Berlin, corporate Member of Freie Universität Berlin and Humboldt-Universität zu Berlin, Department of Cardiology, Hindenburgdamm 30, Berlin 12203, Germany; DZHK (German Centre for Cardiovascular Research), Partner Site Berlin, Berlin, Germany; Deutsches Herzzentrum der Charité, Department of Cardiology, Angiology and Intensive Care Medicine, Campus Benjamin Franklin, Hindenburgdamm 30, Berlin 12203, Germany; Charité—Universitätsmedizin Berlin, corporate Member of Freie Universität Berlin and Humboldt-Universität zu Berlin, Department of Cardiology, Hindenburgdamm 30, Berlin 12203, Germany; Deutsches Herzzentrum der Charité, Department of Cardiology, Angiology and Intensive Care Medicine, Campus Benjamin Franklin, Hindenburgdamm 30, Berlin 12203, Germany; Charité—Universitätsmedizin Berlin, corporate Member of Freie Universität Berlin and Humboldt-Universität zu Berlin, Department of Cardiology, Hindenburgdamm 30, Berlin 12203, Germany; Deutsches Herzzentrum der Charité, Department of Cardiology, Angiology and Intensive Care Medicine, Campus Benjamin Franklin, Hindenburgdamm 30, Berlin 12203, Germany; Charité—Universitätsmedizin Berlin, corporate Member of Freie Universität Berlin and Humboldt-Universität zu Berlin, Department of Cardiology, Hindenburgdamm 30, Berlin 12203, Germany; Deutsches Herzzentrum der Charité, Department of Cardiology, Angiology and Intensive Care Medicine, Campus Benjamin Franklin, Hindenburgdamm 30, Berlin 12203, Germany; Charité—Universitätsmedizin Berlin, corporate Member of Freie Universität Berlin and Humboldt-Universität zu Berlin, Department of Cardiology, Hindenburgdamm 30, Berlin 12203, Germany; DZHK (German Centre for Cardiovascular Research), Partner Site Berlin, Berlin, Germany; Berlin Institute of Health at Charité—Universitätsmedizin Berlin, BIH Biomedical Innovation Academy, Charitéplatz 1, Berlin 10117, Germany; Deutsches Herzzentrum der Charité, Department of Cardiology, Angiology and Intensive Care Medicine, Campus Benjamin Franklin, Hindenburgdamm 30, Berlin 12203, Germany; Charité—Universitätsmedizin Berlin, corporate Member of Freie Universität Berlin and Humboldt-Universität zu Berlin, Department of Cardiology, Hindenburgdamm 30, Berlin 12203, Germany; Institute of Active Polymers and Berlin-Brandenburg Center for Regenerative Therapies, Helmholtz-Zentrum Hereon, Kantstraße 55, Teltow 14513, Germany; Deutsches Herzzentrum der Charité, Department of Cardiology, Angiology and Intensive Care Medicine, Campus Benjamin Franklin, Hindenburgdamm 30, Berlin 12203, Germany; Charité—Universitätsmedizin Berlin, corporate Member of Freie Universität Berlin and Humboldt-Universität zu Berlin, Department of Cardiology, Hindenburgdamm 30, Berlin 12203, Germany; Institute of Active Polymers and Berlin-Brandenburg Center for Regenerative Therapies, Helmholtz-Zentrum Hereon, Kantstraße 55, Teltow 14513, Germany

**Keywords:** Three-dimensional echocardiography, Intracardiac echocardiography, Transcatheter edge-to-edge repair, Tricuspid regurgitation, Local anaesthesia, Case report

## Abstract

**Background:**

Tricuspid transcatheter edge-to-edge repair (T-TEER) effectively reduces tricuspid regurgitation (TR) severity and improves symptoms and functional outcome in patients with TR and high surgical risk. Procedural guidance using intracardiac echocardiography (ICE) is a promising alternative in case of impaired imaging quality or contraindications for transoesophageal echocardiography (TEE) but also to defer any form of anaesthesia. The present case report aims to display the feasibility of an ICE-only guided T-TEER procedure in local anaesthesia and respective set-up.

**Case summary:**

A multimorbid 74-year-old man with severe mixed secondary TR was admitted for elective T-TEER. He suffered from NYHA III dyspnoea, chronic lower leg oedema and had two heart failure hospitalizations in the last 14 months. Local heart team recommended T-TEER due to the high individual surgical risk. TR was successfully reduced to trace by ICE-only guided T-TEER with implantation of two devices, one in anteroseptal and one in posteroseptal position in conventional clover technique. The patient was discharged without any notable complications.

**Discussion:**

The present case report demonstrates the feasibility of an ICE-only guided T-TEER, exclusively conducted in local anaesthesia. High-quality intraprocedural imaging guidance was essential for achieving procedural success.

Learning points

- ICE is a promising alternative to TEE for procedural imaging guidance in T-TEER but has been used almost exclusively in a complementarily manner so far.
- ICE-only guidance of T-TEER is feasible and should be considered in case of limitations and contraindications for TEE.

**Primary specialties involved:** Cardiology, Cardiac surgery, Anaesthesiology (Heart Team)

## Introduction

Moderate or severe tricuspid regurgitation (TR) affects approximately 0.5% of the general population and 4% of people aged over 75.^1^ Severe TR leads to recurrent heart failure hospitalizations (HFH), impaired quality of life and increased mortality.^2–5^ Current ESC guidelines recommend either surgical or interventional treatment of severe TR, depending on the surgical risk.^1^ In case of prohibitive surgical risk, there are currently two concepts of transcatheter therapies available: tricuspid transcatheter edge-to-edge repair (T-TEER) and transcatheter tricuspid valve replacement (TTVR).^2^ T-TEER is known to effectively reduce TR severity, improves quality of life and decreases the need for heart failure hospitalizations.^4,5^ The success of T-TEER procedures relies on imaging guidance with transoesophageal echocardiography (TEE) guidance being state of the art. However, contraindications and technical or anatomical challenges limit its use.^6^ Intracardiac echocardiography (ICE) represents an innovative and promising alternative for procedural guidance.^7^

## Summary figure

**Table:** 

Time	Event	Description
October 2024	Heart failure hospitalization	Exacerbated dyspnoea (NYHA III), ascites, pleural effusion, peripheral oedema, severe tricuspid regurgitation; improved by optimized medical therapy, moderate tricuspid regurgitation (grade 2+) under euvolemic conditions in transthoracic echocardiography
September 2025	Heart failure hospitalization	Exacerbated dyspnoea (NYHA III), ascites, peripheral oedema, stasis dermatitis, severe mixed secondary tricuspid regurgitation (grade 3+) despite guideline directed medical therapy under euvolemic conditions in transoesophageal echocardiography
October 2025	Right heart catherization	Exclusion of pulmonary hypertension (mean pulmonary artery pressure: 18 mmHg, pulmonary artery wedge pressure: 15 mmHg, pulmonary vascular resistance: 1.4 WU, cardiac index: 2.2 L/min/m2)
October 2025	Heart Team	Decision for tricuspid transcatheter edge-to-edge repair
December 2025	Tricuspid transcatheter edge-to-edge repair	Intracardiac echocardiography-only guided transcatheter edge-to-edge repair, severe tricuspid regurgitation reduced to mild (intracardiac echocardiography)
December 2025	Follow-up	Trace tricuspid regurgitation (transthoracic echocardiography) with improved volemic status

## Case presentation

A 74-year-old man (height: 174 cm, weight: 108 kg, BMI: 36 kg/m^2^) was admitted for elective treatment of severe TR with T-TEER. Upon admission, the patient suffered from severe dyspnoea (NYHA III) and mild, chronic leg oedema. The first and second heart sounds were regular, and no pathological heart murmurs were auscultated. The patient was multimorbid and suffered, among other comorbidities, from heart failure with mildly reduced ejection fraction (LVEF: 48%, NT-pro-BNP: 2537 ng/L), persistent atrial fibrillation, arterial hypertension, a history of ischaemic stroke, chronic kidney disease (KDIGO: G2A3), peripheral artery disease (Fontaine IV, Rutherford 5), and liver cirrhosis (CHILD A). Heart failure medication comprised sacubitril/valsartan 24 mg/26 mg twice daily, bisoprolol 10 mg once daily, spironolactone 50 mg once daily, empagliflozin 10 mg once daily, and torasemide 20 and 10 mg once daily. The patient had not undergone any valvular interventions yet.

In the past 14 months, there had already been two heart HFH. After the first decompensation, medical therapy was optimized and moderate TR (grade 2+) was detected by transthoracic echocardiography (TTE) under euvolemic conditions. However, after the second HFH, transoesophageal echocardiography (TEE) revealed severe mixed secondary TR (grade 3+) under euvolemic conditions despite optimized guideline directed medical therapy. Pulmonary hypertension was ruled out by right heart catheterization (mean pulmonary artery pressure: 18 mmHg, pulmonary artery wedge pressure: 15 mmHg, pulmonary vascular resistance: 1.4 WU, cardiac index: 2.2 L/min/m^2^). Due to persistent severe symptomatic TR (grade 3+, vena contracta: 9 mm) despite optimized therapy and high individual surgical risk (TRI-SCORE: 22%, 6/12 points; TRIVALVE-SCORE: 3.5/4.5), the local heart team recommended T-TEER.

**Figure 1:**
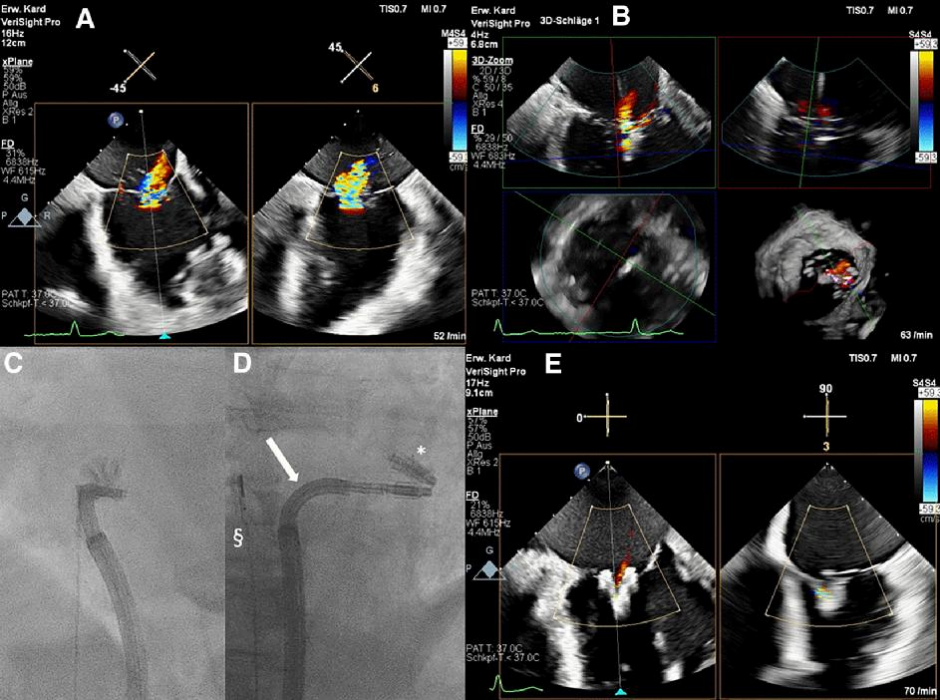
Procedural images by intracardiac echocardiography and conventional fluoroscopy. Legend: Panel *A*): preprocedural biplane imaging with colour doppler showing severe tricuspid regurgitation; Panel *B*): periprocedural multiplanar reconstruction of residual tricuspid regurgitation after implantation of the first device; Panel *C*) and *D*): fluoroscopic imaging describing the anatomical relationship between both devices in en-face [Panel *C*)] and right anterior oblique view [Panel *D*)], *: tricuspid transcatheter edge-to-edge repair device, arrow: device delivery system, §: three-dimensional intracardiac echocardiography probe; Panel *E*): biplane imaging with colour doppler demonstrating the final result with mild residual tricuspid regurgitation after implantation of two devices.

Re-admission to conduct the procedure followed seven weeks later. The procedure was performed without sedation in local anaesthesia only due to exclusive imaging by 3D ICE because of the patient’s multimorbidity and anaesthesiologic risk. The ICE probe (Philips Verisight, Amsterdam, Netherlands) was inserted via the left femoral vein. The main jet of TR was located centrally (biplane vena contracta 9 × 11 mm), beginning in the anteroseptal and extending to the posteroseptal commissure (*Figure 1A*, Supplementary material online, *Video S1*). After puncturing the right femoral vein, the sheath of the T-TEER system was inserted into the right atrium. After placing the first clip in a central position within the anteroseptal commissure (see *Video 1* and Supplementary material online, *Video S2*), there was significant residual regurgitation posteroseptally (*Figure 1B*, Supplementary material online, *Video S3*), so a second clip was placed centrally in the posteroseptal commissure (*Figure 1C*, *Videos 2* and *3*, and Supplementary material online, *Video S4*). Overall, the TR was reduced to a mild TR (grade 1+, *Figure 1D*, Supplementary material online, *Video S5*) without a significant increase in the transvalvular pressure gradient or any procedure-related complications. The TTE performed before discharge showed that both devices were firmly anchored and well positioned, with only minimal residual TR (trace).

## Discussion

The present case report demonstrates that ICE-only guided T-TEER is feasible. TR was significantly reduced without any notable complications. High-quality intraprocedural imaging guidance is essential for achieving success during T-TEER.^8^ TEE guidance is the current gold standard for atrioventricular transcatheter interventions, including T-TEER, recommended by current ESC guidelines.^1^ However, ICE represents a promising alternative for structural heart interventions, especially with the availability of sufficient imaging quality in biplane imaging as well as multiplanar reconstructions, as available with TEE guidance.^9,10^ Both TEE and ICE have distinct advantages and disadvantages for T-TEER guidance (*Table 1*).

**Table 1:** Advantages and disadvantages of transoesophageal echocardiography and intracardiac echocardiography guidance for tricuspid transcatheter edge-to-edge repair

	TEE guidance	ICE guidance
Pro	Gold standard Extensive experience in guiding structural interventions Excellent field of view High resolution imaging Deeper tissue penetration Cost-efficient Preprocedural planning with imaging possible Imaging quality is predictable	No need for anaesthesia or analgosedation Overcoming of certain limitations of TEE (anatomical challenges, shadowing from intracardiac devices/structures, TEE contraindications) High-quality near-field images Single-operator performance or remote approaches possible (if desirable)
Con	Need for anaesthesia or analgosedation Additional personnel required (echocardiocardiographer) Risk of oesophageal injury Compromises in image quality or limitations due to anatomical abnormalities (e.g. poor window, hiatal hernia) Shadowing from intracardiac devices (e.g. mechanical heart valve) and native structures (e.g. calcification) Contraindications (e.g. oesophageal varices)	High costs (single-use catheters, dedicated echocardiography machine required) Limited penetration depth Limited experience, especially in valvular procedures guided by ICE-only Dedicated training and experience required Additional venous access (potential vascular complications) Movement of probe during procedure, may be challenging to maintain a stable view (during respiration, during insertion of other catheters or devices) Navigation of probe during procedure may be challenging due to anatomical causes (e.g. large atria, Chiari’s network, prominent Eustachian valve or Cor triatriatum dexter) Preprocedural planning with imaging not possible in case of contraindications to TEE Imaging quality with ICE is not predictable Artefacts not always excluded (e.g. CIED leads)

CIED, cardiac implantable electronic device; ICE, intracardiac echocardiography; TEE, transoesophageal echocardiography.

**Figure 2:**
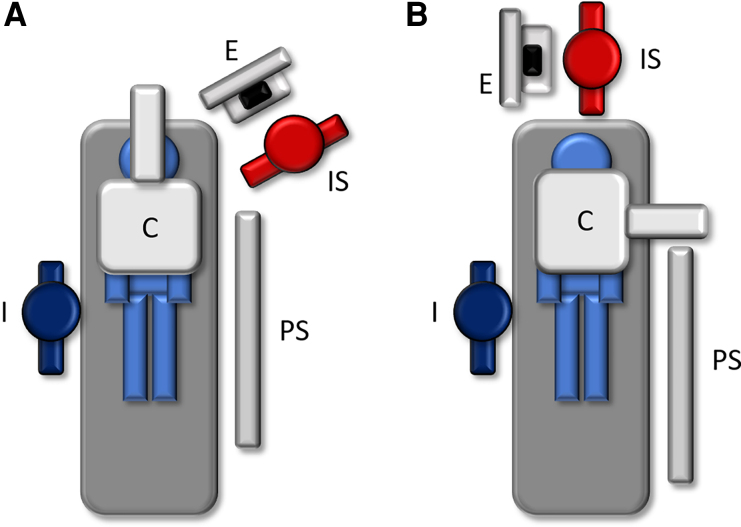
Potential transfemoral setups for single operator intracardiac echocardiography-only guided transcatheter tricuspid edge-to-edge repair. Legend: Panel *A*) shows a setup with cranial introduction of the fluoroscopic C-arm, while panel *B*) describes a setup with left-sided introduction; C: fluoroscopic C-arm, E: echocardiography machine, I: interventionalist, who is manoeuvring the probe and performing T-TEER, IS: imaging specialist, who is processing the delivered imaging by creating 3D multi-planar reconstructions and following the device accordingly, PS: projection screen.

For T-TEER guidance, ICE has mostly been used complementarily to TEE so far.^9^ There are only very few case reports which report on ICE-only guidance for T-TEER procedures.^11,12^ However, due to absolute contraindications, there are also some clinical scenarios in which TEE guidance is not possible at all (e.g. oesophageal varices or recent upper gastrointestinal surgery). Moreover, ICE guidance has numerous further potential advantages. Since recent imaging quality enhancements due to improved technology allow ICE to be used as only echocardiographic imaging modality, the procedure may be guided exclusively via intravascular access without any anaesthesia or analgosedation required. This could increase procedural workflows by eliminating anaesthesia induction and weaning times as well as overcome the potential limitation of unavailability of dedicated anaesthesiologists in some centres. Beyond, the entire procedure can be performed by a single operator as shown by case reports performed in left atrial appendage occlusion.^13^ Potential transfemoral setups are displayed in *Figure 2*. ICE guidance may also be performed via jugular venous access. However, conventional fluoroscopy can make an important contribution to the alignment of the clip in relation to the respective commissures. The technical possibility of fluoroscopy and ICE fusion imaging is already available.

However, high costs and a greater learning curve currently prevent widespread use of ICE guidance. As a result, only a few experienced centres currently have sufficient experience with ICE guidance. Moreover, additional vascular complications may occur at the additional vascular access, and the ICE probe may need to be stabilized during the procedure. In some views, the field of view is also limited, and the image quality is inferior.^8,9,14^

In conclusion, presented case describes the feasibility of ICE-only guided T-TEER procedures. In patients with contraindications to TEE, ICE-only guidance should be considered. Future trials are warranted to weigh its potential upsides against the downsides.

## Supplementary Material

ytag466_Supplementary_Data

## Data Availability

The data underlying this article are available in the article.

## Lead author biography

Lukas Ley works as a cardiology resident at Deutsches Herzzentrum der Charité at Campus Benjamin Franklin in Berlin (DE). He graduated from Justus-Liebig-University in Giessen (DE) with study periods in Cape Town (ZA), London (GB), and Baden (CH). He has particular clinical and scientific interest in valvular heart disease, especially tricuspid regurgitation, right ventricular function, and pulmonary hypertension.
